# CCL18 aggravates atherosclerosis by inducing CCR6-dependent T-cell influx and polarization

**DOI:** 10.3389/fimmu.2024.1327051

**Published:** 2024-05-13

**Authors:** Anjana Singh, Adriaan O. Kraaijeveld, Adelina Curaj, Kanin Wichapong, Linda Hammerich, Saskia C. A. de Jager, Ilze Bot, Sergei P. Atamas, Theo J. C. van Berkel, J. Wouter Jukema, Iain Comerford, Shaun R. McColl, Barend Mees, Johan W. M. Heemskerk, Gerry A. F. Nicolaes, Tilman Hackeng, Elisa Anamaria Liehn, Frank Tacke, Erik A. L. Biessen

**Affiliations:** ^1^ Department of Pathology, Cardiovascular Research Institute Maastricht (CARIM), Maastricht University, Maastricht, Netherlands; ^2^ Leiden Academic Center for Drug Research, Leiden University, Leiden, Netherlands; ^3^ Department of Cardiology, University Medical Center Utrecht, Utrecht, Netherlands; ^4^ Institute for Molecular Cardiovascular Research, Rheinisch-Westfälische Technische Hochschule (RWTH) Aachen, Aachen, Germany; ^5^ Department of Biochemistry, Cardiovascular Research Institute Maastricht, Maastricht University, Maastricht, Netherlands; ^6^ Department of Experimental Cardiology, University Medical Center Utrecht, Utrecht, Netherlands; ^7^ Department of Medicine, University of Maryland School of Medicine, Baltimore, MD, United States; ^8^ Department of Cardiology, Leiden University Medical Center (LUMC), Leiden, Netherlands; ^9^ Centre for Molecular Pathology, School of Molecular & Biomedical Science, University of Adelaide, Adelaide, SA, Australia; ^10^ Department of Surgery, Maastricht University, Maastricht, Netherlands; ^11^ Department of Hepatology & Gastroenterology, Charité - Universitätsmedizin Berlin, Berlin, Germany

**Keywords:** chemokines, inflammation, Th17, plaque, cardiovascular, chemotaxis

## Abstract

**Introduction:**

The CC chemokine ligand 18 (CCL18) is a chemokine highly expressed in chronic inflammation in humans. Recent observations of elevated CCL18 plasma levels in patients with acute cardiovascular syndromes prompted an investigation into the role of CCL18 in the pathogenesis of human and mouse atherosclerosis.

**Methods and results:**

CCL18 was profoundly upregulated in ruptured human atherosclerotic plaque, particularly within macrophages. Repeated administration of CCL18 in Western-type diet–fed *ApoE* ^−/−^ mice or PCSK9^mut^-overexpressing wild type (WT) mice led to increased plaque burden, enriched in CD3^+^ T cells. In subsequent experimental and molecular modeling studies, we identified CCR6 as a functional receptor mediating CCL18 chemotaxis, intracellular Ca^2+^ flux, and downstream signaling in human Jurkat and mouse T cells. CCL18 failed to induce these effects *in vitro* in murine spleen T cells with CCR6 deficiency. The ability of CCR6 to act as CCL18 receptor was confirmed *in vivo* in an inflammation model, where subcutaneous CCL18 injection induced profound focal skin inflammation in WT but not in *CCR6^−/−^* mice. This inflammation featured edema and marked infiltration of various leukocyte subsets, including T cells with a Th17 signature, supporting CCR6’s role as a Th17 chemotactic receptor. Notably, focal overexpression of CCL18 in plaques was associated with an increased presence of CCR6^+^ (T) cells.

**Discussion:**

Our studies are the first to identify the CCL18/CCR6 axis as a regulator of immune responses in advanced murine and human atherosclerosis.

## Introduction

Atherosclerosis is a chronic inflammatory disease characterized by arterial lesions consisting of cholesterol and connective tissue deposits and immune cell infiltrates (1, 2), which is guided by chemokines (3). Recent studies have considerably increased our understanding of chemokine mediated leukocyte recruitment and responses, including differentiation, activation (4), apoptosis (5), development (6), and angiogenesis (7). Chemokines act through interaction with seven-transmembrane domain G protein–coupled receptors (GPCRs) on their target cells (2). Several chemokines have so far been implicated in experimental atherosclerosis and in cardiovascular disorders (3, 8), although causality remains to be established for several of these associations. For instance, we and others have previously shown plasma levels of CC chemokine ligand 18 (CCL18), a chemokine with presumed constitutive expression in lung and blood, to be elevated in persistent unstable angina pectoris and to correlate with future cardiovascular events (8, 9). Moreover, CCL18 was seen to be expressed in atherosclerotic plaques, particularly in areas of decreased stability (10), where it co-localizes with CD83+ dendritic cells. For long viewed as orphan ligand, CCL18 was recently proposed not only to be a competitive inhibitor of CCL11 (eotaxin) binding to chemokine receptor 3 (CCR3) (11) but also to bind to CCR8 (12), phosphatidylinositol transfer protein 3 (PITPNM3) (13), nitrite reductase 1 (NIR1) (14), and CCR6 (15), although a subsequent study failed to confirm this link (16). CCL18 is produced by monocyte subsets and naïve CD4^+^ T cells and able to stimulate collagen production by fibroblasts (17). CCL18 is highly expressed in many human chronic inflammatory diseases, such as Th2 pulmonary fibrosis, where it contributes to disease progression (18). On the basis of its expression in atherosclerotic plaque and the reported T-cell chemotactic activity, we postulated that CCL18 is causally implicated in the pathophysiology of atherosclerosis. Our studies reveal a pro-atherogenic role for CCL18 *in vitro* and *in vivo* and identify CCR6 as *bona fide* receptor for CCL18, mediating chemotaxis of T cells and augmenting plaque immune responses. Our work thus provides evidence and a molecular foundation for its causal involvement in atherosclerosis and paves the way for CCL18-targeted treatment measures.

## Materials and methods

### Human plaque expression studies

Total RNA was extracted from freshly frozen atherosclerotic tissue samples obtained from endarterectomy surgery [comparison advanced stable and advanced unstable lesions; Maastricht Human Plaque Study-1 (MHPS1) cohort; n = 43] or from autopsy [comparison early (n = 13) and advanced stable (n = 16) lesions; Department of Pathology, University Hospital Maastricht, Maastricht, The Netherlands]. Collection, storage in the Maastricht Pathology Tissue Collection, and patient data confidentiality as well as tissue usage were in accordance with the “Code for Proper Secondary Use of Human Tissue in The Netherlands.” Tissue handling and analyses conformed to the principles as outlined in the Declaration of Helsinki. Tissue samples destined for RNA isolation were snap-frozen immediately after resection, staged by histological analysis of adjacent tissue sections according to Virmani et al. (19) and grouped as advanced stable (fibrous cap atheroma (FCA) or pathological intimal thickening) or advanced unstable lesions (if featuring an intraplaque hemorrhage or intramural thrombus). Lesions flanked at both sides by sections of a stable phenotype (based on morphological assessment) were considered stable and *vice versa*. RNA was isolated with the Guanidine Thiocyanate/CsCl gradient method and the NucleoSpin RNA II kit (Macherey-Nagel GmbH & Co. KG). RNA concentration was determined using the Nanodrop ND-1000 (Thermo Scientific), and quality was assessed by RNA 6000 Nano/Pico LabChip (Agilent 2100 Bioanalyzer, Palo Alto, CA, USA) analysis based on RIN (RNA integration number) values. RIN values above 5.6 were considered acceptable. Samples from autopsy were individually hybridized to HGU133 2.0 Plus arrays (Affymetrix, Santa Clara, USA, California); surgery samples were analyzed by Illumina Human Sentrix-8 V2.0 BeadChip^®^ (Illumina Inc., San Diego, USA, California).

### Animal studies

All animal work, described in detail in the supplemental methods section, was approved by the local regulatory authority of Maastricht University, and procedures conformed to the guidelines from Directive 2010/63/EU of the European Parliament on the protection of animals used for scientific purposes. Unless otherwise stated, animal experiments were performed under anesthesia [subcutaneous injection of ketamine (60 mg/kg), fentanyl citrate (1.3 mg/kg), and fluanisone (2 mg/kg)]; mice were euthanized at the end of the experiment by cervical dislocation under anesthesia.

### *In vivo* kinetics of CCL18

CCL18 was radiolabeled with ^125^I (GE Healthcare, Diegem, Belgium) according to the Iodogen method (20). To examine its *in vivo* kinetics after repeated administration, ^125^I-CCL18 was administered once daily to C57Bl6 mice (Charles River, Maastricht, The Netherlands) by intraperitoneal administration (doses of 2.5 μg and 5 μg); at 1 h, 4 h, and 24 h after each injection, blood samples were taken and counted for radioactivity. Mice were euthanized at 72 h following the first injection, main organs and tissues were excised, and ^125^I accumulation was counted by a Wizard 1470 automatic gamma counter (Perkin Elmer, Waltham, MA).

### Intradermal CCL18 injection

We performed intradermal injection of 20 μL of phosphate buffered saline (PBS), either alone or containing 500 ng of recombinant mouse CCL18 (SeroTech), into the ears of anesthetized mice using a 30-gauge needle every other day for 12 days. Ear thickness was measured before injection on day 0 and, thereafter, on non-injection days. Ear measurements were made using a caliper (GEM TOOL - Digital Thickness Gauge, 0–25 mm). All measurements were performed blinded. Mice were sacrificed at day 2 and day 12, and tissue was collected for histological, flow cytometric, and expression analysis.

### Effect of systemic CCL18 administration on atherosclerosis

Effects of systemic administration of CCL18 on pre-existing atherosclerosis were studied in *ApoE* ^−/−^ mice (female; 10–12 weeks of age), fed with a Western-type diet (WTD) containing 0.25% cholesterol and 15% cocoa butter (Special Diets Services, Witham, Essex, UK). Female mice were used as they show reduced variability in lesion formation compared with male mice. Diet and water were provided *ad libitum*. After 6 weeks of WTD, mice received an initiation bolus of CCL18 (5 μg) in PBS/0.5% bovine serum albumin (BSA) (100 μL) via intraperitoneal injection, followed by two daily injections of CCL18 (2.5 μg) for 2 weeks. As controls, mice were injected with PBS/0.5%BSA (100 μL). After isolation, the aortic arch and heart were fixed overnight in 1% paraformaldehyde (PFA), and paraffin-embedded sections (5 μm) were cut, starting from the commissure of the aortic cups upward. Plaque size and necrotic core content in the aortic root were determined by morphometric analysis of the sections and by averaging the mean lesion area of 10 consecutive sections by Leica QWin software.

### Focal overexpression of CCL18 in pre-existing plaques

*ApoE* ^−/−^ mice (n = 24) (female, 10–12 weeks) were obtained from our own breeding stock and were put on a WTD. After 2 weeks, mice received constrictive collars around both carotid arteries as previously described (21). At this point, collars were removed, the internal carotid artery and the common carotid proximal to the plaque were clamped, and the adenovirus suspension (Ad.-CCL18-GFP and Ad-Empty-GFP, respectively, at a titre of 1.0 × 10^10^ plaque forming units (pfu)/mL; 20 μL) was instilled via the left common carotid artery and left to incubate for 10 minutes. Virus load for all treatment groups was equal and was tolerated well, with no detectable endothelial inflammatory effects. Two weeks after local incubation, the mice were sacrificed; before harvesting, the arterial bed was flushed for 10’ with PBS/p-formaldehyde (4%). Fixated carotid arteries were embedded in optimum cutting temperature (OCT) (Sakura Finetek, Zoeterwoude, The Netherlands), snap-frozen in liquid nitrogen, and stored at −20°C until transverse cryosections were prepared (5 µm).

### Recombinant AAV-PCSK-9 gene delivery into the WT and *CCR6* ^−/−^ mice

WT and *CCR6* ^−/−^ littermates (females, 12–14 weeks of age) were injected intravenously with AAV-PCSK-9 (5 × 10^10^ pfu) and challenged with WTD for 6 weeks (22). After 6 weeks of WTD, WT and *CCR6* ^−/−^ mice (n = 6) received an initiation bolus of 5 μg of CCL18 in 100 μL of PBS/0.5%BSA via intraperitoneal injection, followed by two daily injections of 2.5 μg of CCL18 for 2 weeks. As a control, mice were injected with 100 μL of PBS/0.5%BSA. Blood samples were collected before and every other week after vector injection to monitor plasma cholesterol.

### Cellular response on CCL18

Jurkat T cells were used to examine proliferation and gene expression after incubation with soluble CCL18. In brief, cells were plated in 24-well plates and grown to 70% confluence in Dulbecco’s modified Eagle medium (DMEM) supplemented with 10% fetal calf serum (FCS), streptomycin (100 μg/mL), penicillin (100 U/mL), and 2 mM L-glutamine. Afterward, cells were serum-starved in DMEM + 1% FCS for 10 h and subsequently incubated for 16 h with CCL18 (30 ng/mL), together with ^3^H-thymidine (925 GBq/mmol; Amersham, Uppsala, Sweden). The next day, ^3^H-thymidine incorporation was measured by a Packard 1500 liquid scintillation analyzer (PerkinElmer), and cells were harvested for RNA isolation. Guanidium thiocyanate-phenol was used to extract total RNA from cells, and samples were subjected to Deoxyribonuclease (DNAse) I treatment (Promega, Madison, WS) after which cDNA was generated using RevertAid M-MuLV reverse transcriptase (Fermentas, Burlington, Canada) according to manufacturer’s protocol. Semi-quantitative gene expression analysis of Interleukin (IL)-2, IL-6, IL-10, IL-15, C-C chemokine receptor type (CCR)3, CCR5, cluster of dirrentiation-40 (CD-40), CD-69, transforming growth factor (TGF-β), interferon (IFN-γ), inducibe NO synthase (i-NOS), liver X-receptor (LXR), and Cytochrome P450 (CYP-7α) was performed using the SYBR-Green method on a FAST 7500 RT-PCR apparatus (Applied Biosystems, Foster City, CA).

### CCL18 peptide synthesis

CCL18 peptide (Ala21 to Ala89) was synthetized by standard solid phase Fmoc chemistry. Purity and identity of the peptide were established by liquid chromatography–mass spectrometry (LC-MS) (Molecular weight (Mw): 7,855).

### CCL18 adenovirus

Adenovirus-expressing human CCL18 under a Cytomegalovirus (CMV) promoter or an empty transcript, labeled with green fluorescent protein (GFP), was generated, produced, and titred as described previously (23).

### *In vivo* CCL18 intervention studies

Details on the *in vivo* atherosclerosis and ear swelling experiments are provided in the supplemental methods.

### Statistical analysis

For microarrays, fold change expression between groups was calculated via the Rosetta Resolver error model (Rosetta Biosoftware). All values are expressed as mean ± SEM, when appropriate. Analyses were done using INSTAT (GraphPad Software). Two-group comparisons were analyzed by Welch corrected Student’s t-test to account for unequal variances, except for higher powered data sets (n > 8) with equivalent variance, where we opted for unpaired t-test. Multi-group comparisons were analyzed by one-way ANOVA, with Kruskal–Wallis post-testing. Two-sided P-values of >0.05 were considered significant and denoted with one (<0.05), two (<0.01), or three asterisks (<0.001).

## Results

### Involvement of the CCL18 in human atherosclerotic lesions

**Figure 1:**
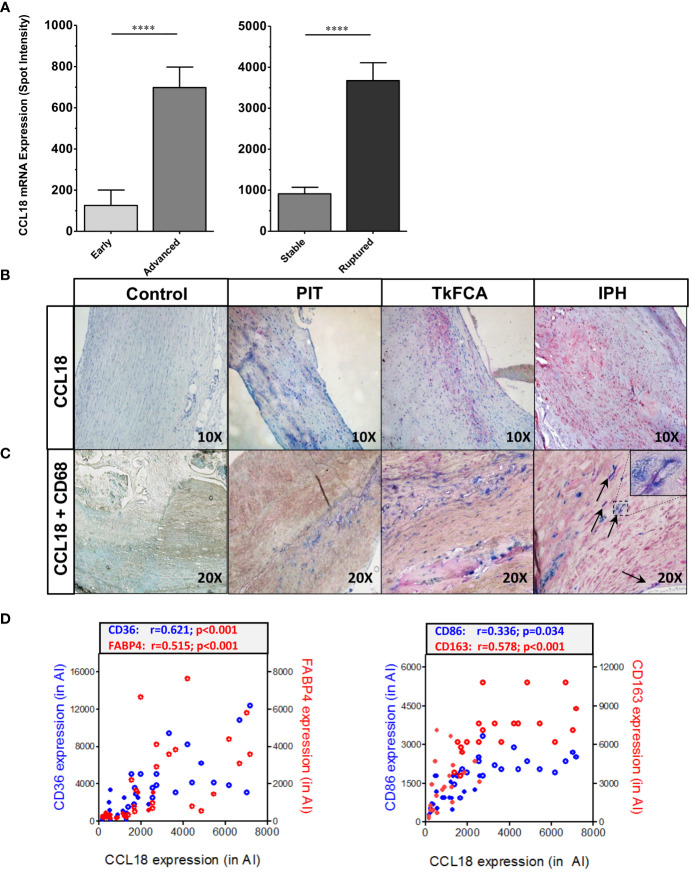
CCL18 expression is expressed in human plaque. **(A)** Microarray analysis of CCL18 expression in early (PIT) vs. advanced (TkFCA) from autopsy (n = 8) and advanced (TkFCA) vs. ruptured (IPH) (n = 23) carotid atherosclerotic lesions obtained by endarterectomy surgery. **(B)** CCL18 expression is progressively upregulated with disease progression (upper panels, 10×). **(C)** CCL18 (red) is abundant in CD68^+^ macrophages (blue) in advanced and ruptured lesions (lower panels, 20×), albeit that not all CD68+ cells and some CD68− cells (possibly fibroblasts) stained positive for CCL18. **(D)** CCL18 *mRNA* correlates with established macrophage markers such as CD36 (blue), FABP4 (red) (both lipid laden macrophages; left panel), CD68 (all macrophages, blue), and CD163 (heme-laden macrophages; red) (both right panel); open symbols refer to ruptured plaques, and closed symbols refer to stable plaques; ****P<0.0001.

CCL18 expression was assessed in diseased human endarterectomy specimens (the MHPS1). Plaques were staged as advanced stable or advanced ruptured according to Virmani’s classification scheme (20). Omics sections were considered of stable or ruptured, if bilaterally flanked by sections of identical phenotype, and were analyzed for gene expression. CCL18 expression was increasingly upregulated with disease progression and was most pronounced in ruptured plaque (**Figure 1A**). This was confirmed by immunohistochemistry, with poor staining in early [pathological intimal thickening (PIT)] and abundant expression in advanced progression stages [intraplaque hemorrhage (IPH)] (**Figure 1B**). CCL18 expression was seen to colocalize with CD68^+^ macrophage staining, albeit that not all CD68+ cells stained positive for CCL18 and that some CD68- negative cells, possibly fibroblasts, were also showing CCL18 staining (**Figure 1C**). In agreement, plaque CCL18 expression showed a significant correlation with that of established macrophage markers, such as CD36 (lipid-laden macrophages), FABP4 (foam cells), CD163 (heme-laden macrophages), and CD68 (all macrophages) (**Figure 1D**).

### CCL18 aggravates atherosclerotic lesion formation.

**Figure 2:**
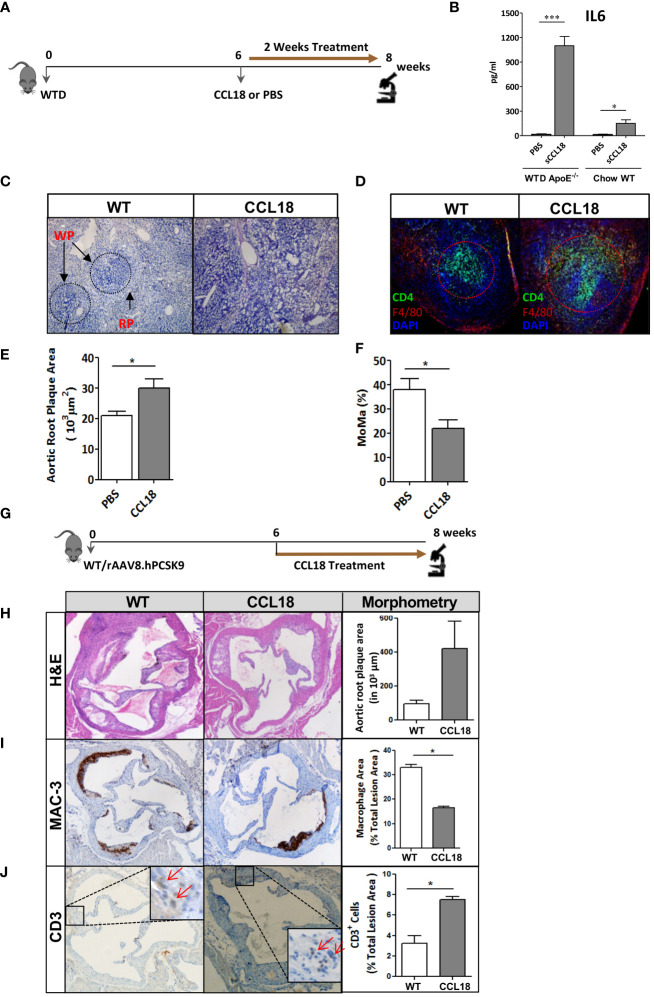
CCL18 treatment significantly increases plaque development in *ApoE*−/− mice. **(A)** Experimental setup. **(B)** Plasma levels of IL-6 in WTD-fed *ApoE* ^−/−^ but not in chow-fed WT mice are significantly increased after CCL18 treatment vs. control (PBS treatment). **(C)** Representative micrographs of Hematoxylin (HE)-stained spleen sections show altered splenic architecture upon CCL18 treatment. Arrows indicate white (WP) and red pulp (RP). **(D)** Representative immunohistochemistry of spleen of mock- vs. CCL18-treated mice (CD4^+^ T cell in green; F4/80+ macrophages in red; 4,6-diamidino-2-phenylindole (DAPI) nuclear counterstain in blue). CCL18-treated mice show disturbed germinal center architecture with more diffuse T-cell distribution. **(E)** Two weeks of CCL18 treatment increased plaque burden in carotid artery of WTD-fed *ApoE* ^−/−^, which was accompanied by **(F)** reduced plaque macrophage presence (relative MOMA^+^ plaque area) (mean ± SEM; n = 8). **(G)** Experimental design. **(H)** Aortic root plaque size of mock- versus CCL18-treated *ApoE* ^−/−^ mice. **(I)** Immunohistochemistry of aortic roots for MAC3 (macrophages) and **(J)** CD3 (T cells). Values are mean ± SEM; n = 6–7 (IHC), n = 8 (IL-6; log-transformed), and n = 12 mice per group (plaque morphometry). *P < 0.05 and ***P < 0.001.

Next, we studied whether human-like CCL18 plasma levels would impact atherosclerotic plaque progression in WTD-fed *ApoE* ^−/−^ mice. For study design see **Figure 2A**. Intraperitoneally injected CCL18 displayed a plasma half-life of approximately 10 h and two daily injections of 2.5 µg/kg resulted in CCL18 plasma levels ranging from 20 ng/mL to 60 ng/mL, similar to levels observed in humans (30–50 ng/mL) (**Supplementary Figure 1A**). Two weeks of CCL18 treatment affected neither plasma cholesterol and triglyceride levels nor body weight (data not shown). As reported (20), CCL18 led to altered serum cytokine profiles, with sharply elevated IL-6 (**Figure 2B**) but not CCL2 or CCL3 levels (**Supplementary Figures 1B, C**). The distribution pattern of the major leukocyte populations in blood remained unchanged after CCL18 treatment (**Supplementary Figure 1D**). Of note, CCL18-treated mice had disorganized germinal centers (**Figure 2C**), with more dispersed CD4^+^ T-cell localization and abundant F4/80 cell presence in the marginal zones (**Figure 2D**). Spleen weight and neutrophil content were unaffected (data not shown).

Regarding atherosclerosis, CCL18 treatment deteriorated plaque formation in WTD-fed *ApoE* ^−/−^ mice. Despite the brief 2-week treatment span, CCL18-treated mice displayed 40% increased plaque burden compared with control-treated mice (**Figure 2E**). Plaques from CCL18-treated mice had decreased macrophage content, compatible with a more advanced plaque phenotype (**Figure 2F**) and tended to have increased collagen content, which is in line with CCL18’s reported pro-fibrotic activity in lung inflammation (18). The pro-atherogenic effects of CCL18 could be confirmed in WTD-fed WT mice, which were rendered low-density lipoprotein receptor (LDLr)-deficient, thus hyperlipidemic, by AAV.PCSK9 treatment (**Figure 2G**). In this case, CCL18 administration also led to plaque expansion, albeit at borderline significance (**Figure 2H**). Plaques of CCL18-treated mice contained significantly fewer MAC-3^+^ macrophages and were enriched in CD3^+^ T cells (**Figures 2I, J**).

### CCL18 activates T cells in G protein–coupled receptor–dependent manner

**Figure 3:**
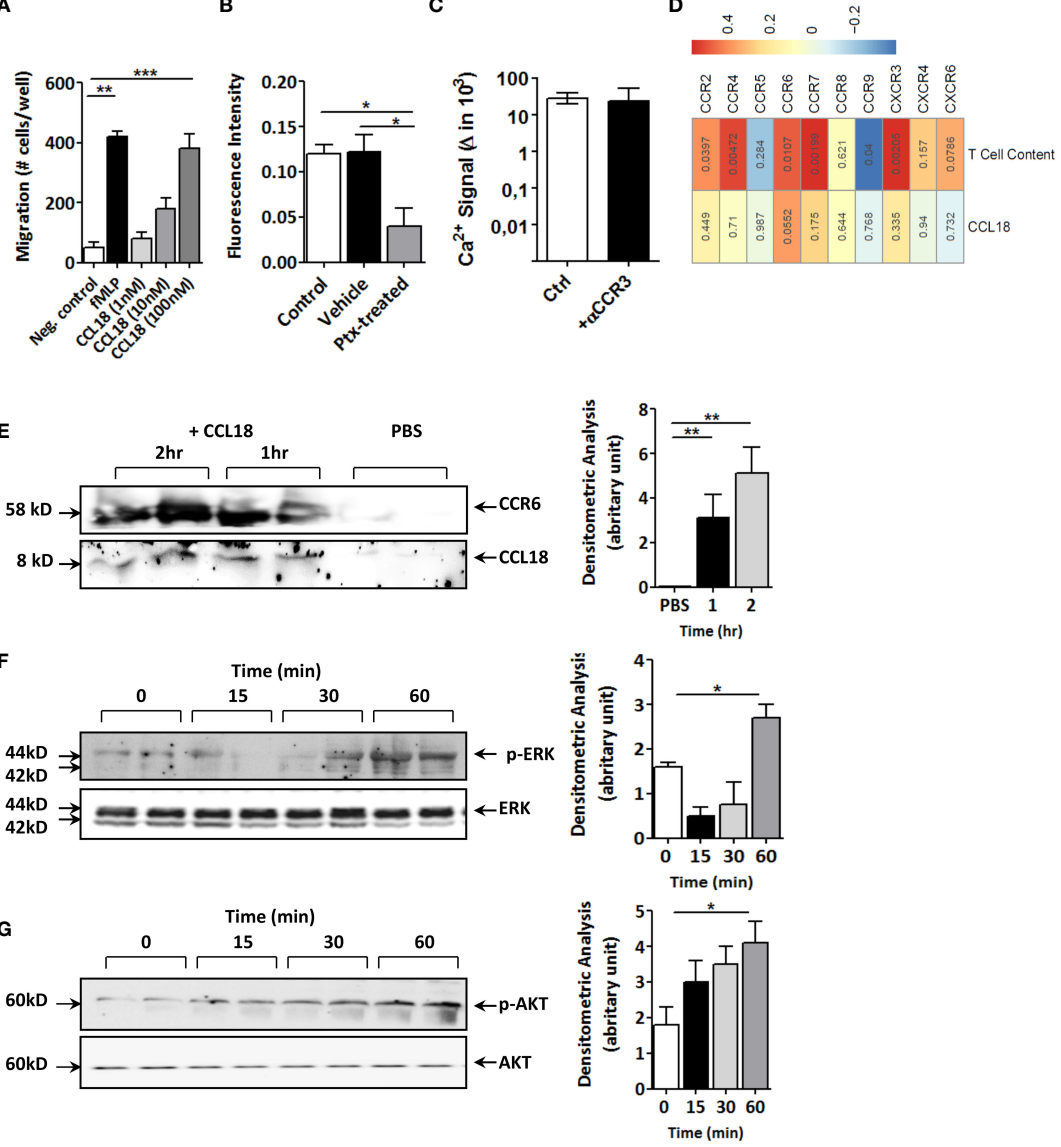
CCL18 induces migration and activation of Jurkat T cells and coprecipitates with CCR6. **(A)** Boyden chamber study of Jurkat T-cell chemotaxis to CCL18 (1–100 nM); fMLP (1 nM) served as reference (n = 4). **(B)** Ca^2+^ mobilization in Jurkat T cells in response to CCL18 (100 nM) is inhibited by pretreatment with pertussis toxin (PTx; 100 ng/mL; n = 4). **(C)** intracellular Ca^2+^ mobilization in Jurkat T cells in response to CCL18 (100 ng/mL) is not inhibited by neutralizing anti-CCR3 (5nM) (n = 4). **(D)** Heatmap showing Pearson correlations of CCL18 expression (lower lane) or plaque T-cell content (upper lane) versus expression of T-cell expressed chemokine receptors for unstable human carotid artery plaques (n = 23, Maastricht Human Plaque Study-1; numbers in cell indicate significance of correlation). **(E)** Coprecipitation of CCL18 (8 kDa) and CCR6 (58 kDa) in CCL18- but not in PBS-incubated Jurkat T-cell lysates. **(F, G)** Western blotting for phosphorylated and total ERK **(F)** and AKT **(G)** of CCL18-stimulated Jurkat T cells at 0 min, 15 min, 30 min, and 60 min after stimulus. Quantification of Western blot bands was done by densitometry (right panels). Presented data are mean ± S.E.M.; *P < 0.05, **P < 0.01, and ***P < 0.001.

Next, we set out to dissect the molecular pathways responsible for this effect. CCL18 incubation led to fivefold increased T-cell proliferation (**Supplementary Figure 1E**) and dose-dependently enhanced T-cell migration *in vitro* (**Figure 3A**), confirming CCL18’s T-cell chemotactic activity (22). While considering Gα_i_ protein–coupled receptors as potential candidates for CCL18 signaling, CCL18, indeed, was able to trigger intracellular Ca^2+^ mobilization in human Jurkat T cells, an effect that was inhibited after pre-incubation with the Gi/o signaling inhibitor pertussis toxin (PTx) (**Figure 3B**). Of the candidate Gα protein–coupled chemokine receptors, CCR3 has previously been linked to CCL18 (22). However, like the isotype IgG control, also CCR3 blocking antibody failed to interrupt the strong CCL18-induced Ca2+ mobilization in Jurkat T cells (**Figure 3C**).

Having excluded CCR3, we interrogated other T-cell expressed chemokine receptors, including the recently postulated candidate receptor for CCL18, notably CCR8 (12). For this, we mapped the correlation of chemokine receptor expression and CCL18 in our cohort of unstable carotid artery plaques (n = 43; MHPS1). CCR8 was virtually undetectable in human atherosclerotic plaque, and its expression did not correlate with that of CCL18 (**Figure 3D**). Of the chemokine receptors tested, only CCR6 showed borderline significant correlation with CCL18 (p = 0.055); its expression was highly correlated with T-cell presence. Similar results were obtained for the whole-plaque cohort (stable and unstable plaque), where CCR6 showed a highly significant (P = 0.001) correlation with CCL18 levels (data not shown) when taking all plaques. Therefore, we considered CCR6, known to display T-cell chemotactic activity and, similar to CCL18, to be implicated in tissue fibrosis (19). Immunoprecipitation of Jurkat T-cell lysates with monoclonal anti-CCL18 antibody in subsequent blotting for CCR6 revealed a 58-kDa protein band, which became more intense with increasing incubation time. Re-analysis for CCL18 showed an 8-kDa protein, immune-reactive for CCL18 in CCL18- but not PBS-treated cell lysates (**Figure 3E**). In keeping with previous reports on CCL20 (24), CCL18 activation of Jurkat T cells was accompanied by an initial decline followed by an increase in ERK phosphorylation (**Figure 3F**), whereas AKT phosphorylation was progressively increased with time (**Figure 3G**). Of note, stimulation of Jurkat T cells with CCL20 decreased phosphorylation of ERK, but unlike CCL18, not of AKT (**Supplementary Figures 2A, B**), suggesting divergent signaling routes for these two chemokines.

**Figure 4:**
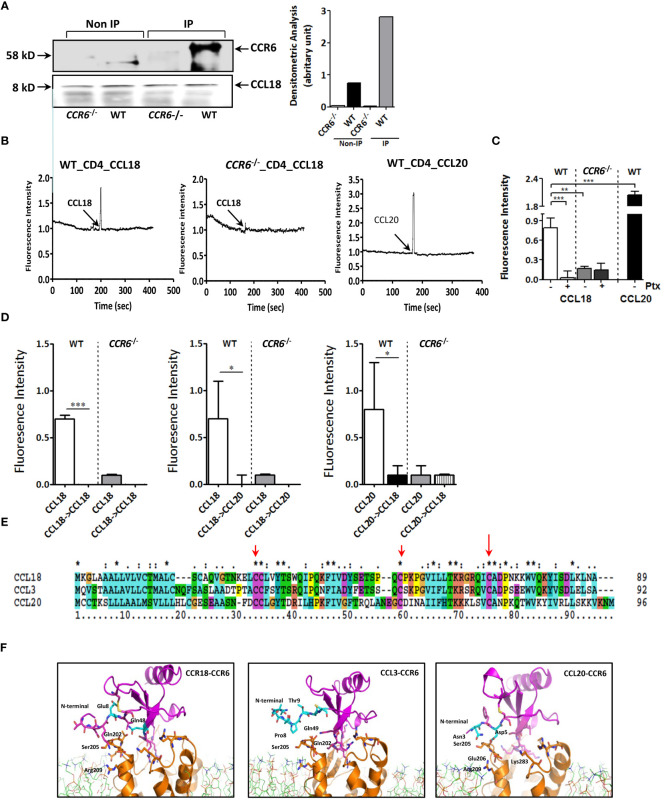
CCL18-induced activation of T cells is dependent on expression of CCR6. **(A)** Splenocytes were treated with CCL18 (100 ng/mL). Immunoprecipitation with anti-CCL18 was performed, followed by non-denaturating gel and Western blot for CCR6 (upper, 58 kDa). Quantification of Western blot bands by densitometry. The CCL18 signal was used as a loading control and for normalization (lower). **(B)** Ca^2+^ mobilization in WT (left and right panels) and *CCR6* ^−/−^ splenic CD4^+^ T cells (middle panel) in response to CCL18 (100 ng/mL; left and middle panel) and CCL20 (30 ng/mL; right panel). **(C)** Ca^2+^ mobilization in mock- and Ptx-treated (100 ng/mL) WT and *CCR6* ^−/−^ splenic CD4^+^ T cells in response to CCL18 (100 ng/mL) and CCL20 (30 ng/mL) (mean ± SEM; n = 4) **(D)** Heterologous desensitization of splenic WT but not *CCR6* ^−/−^ CD4^+^ T cells in response to CCL18 and CCL20; after priming with CCL18 (100 ng/mL; left and middle panel) or CCL20 (30 ng/mL; right panel), cells were washed, restimulated with CCL18 (100 ng/mL; left and right panel) or CCL20 (10 ng/mL; middle panel) and intracellular Ca^2+^ levels were measured (mean ± SEM; n = 4); **(E)** Amino acid alignment of CCL18 with human CCL20 and CCL3. **(F)** Molecular dynamics stimulation showing the predicted CCR6 residue interactions with CCL18, CCL20, and CCL3. *P < 0.05, **P < 0.01, and ***P < 0.001.

CCL18 and CCL20 did not affect baseline apoptosis of Jurkat T cells as judged by Annexin-A5 flow cytometry (**Supplementary Figure 2C**). Western blotting analysis confirmed the almost complete CCR6 deletion in *CCR6* ^−/−^ mice (**Supplementary Figure 2D**). Concordant with our human data, anti-CCL18 antibody immunoprecipitated from mouse splenocyte lysates showed a pronounced 58-kDa protein band in WT but not in *CCR6* ^−/−^ splenocytes, as well as an 8-kDa protein, immune-reactive for CCL18 (**Figure 4A**). At the functional level, intracellular Ca^2+^ levels were rapidly increased upon CCL18 stimulation of WT but not *CCR6* ^−/−^ CD4^+^ splenic T cells. CCL20, the canonical CCR6 ligand induced robust Ca^2+^ mobilization in CD4^+^ T cells of WT but not *CCR6* ^−/−^ mice, as expected (**Figure 4B**). PTx abrogated CCL18 elicited Ca^2+^ mobilization in WT but not in *CCR6* ^−/−^ CD4^+^ T cells, identifying CCR6 as main CCL18-responsive Gα_i_ protein–coupled receptor on splenocytes (**Figure 4C**). Heterologous desensitization of ligand induced Ca^2+^ mobilization is routinely used to pinpoint a shared receptor. Pre-stimulation of WT CD4^+^ T cells with CCL18 led to blunted Ca^2+^ signaling response to CCL20 and *vice versa*; whereas, in *CCR6* ^−/−^ CD4^+^ T cells, CCL18 completely failed to induce Ca^2+^ influx. These results demonstrate that, analogous to CCL20, CCL18 causes full CCR6 desensitization (**Figure 4D**).

Interestingly, amino acid sequence alignment of CCL18, CCL3, and CCL20 indicated high homology of CCL18 with CCL3 (60.9% identical amino acids) and a moderate one with CCL20 (25.5%) (**Figure 4E**). Structure analysis by computer-assisted molecular dynamic (MD) modeling revealed that, whereas CCL3 and CCL18 shared typical CC-chemokine structural features, CCL18 differed from CCL3 in the flexible amino-terminal domain preceding the CC motif, which is instrumental in receptor binding (24). MD simulations of CCR6/CCL18 versus CCR6/CCL3 binding confirmed a differential interaction between the N-termini of CCL3, CCL18, and CCL20 and CCR6 extracellular loops (**Figure 4F**). More specifically, our data showed clear interaction between Glu8 in CCL18 and Ser205/Arg209 in CCR6, mirroring that of CCL20 N-terminal domain residues Asn3 and Asp5 with Ser205/Glu206 and Lys83 in CCR6. The N-terminus of CCL3, however, was unable to bind the CCR6 extracellular loops, likely because the CCL3 N-terminus lacks negatively charged residues, deemed important for guiding and stabilizing the aforementioned interactions.

### CCR6 is required for CCL18-induced leukocyte recruitment and chronic inflammation *in vivo*

**Figure 5:**
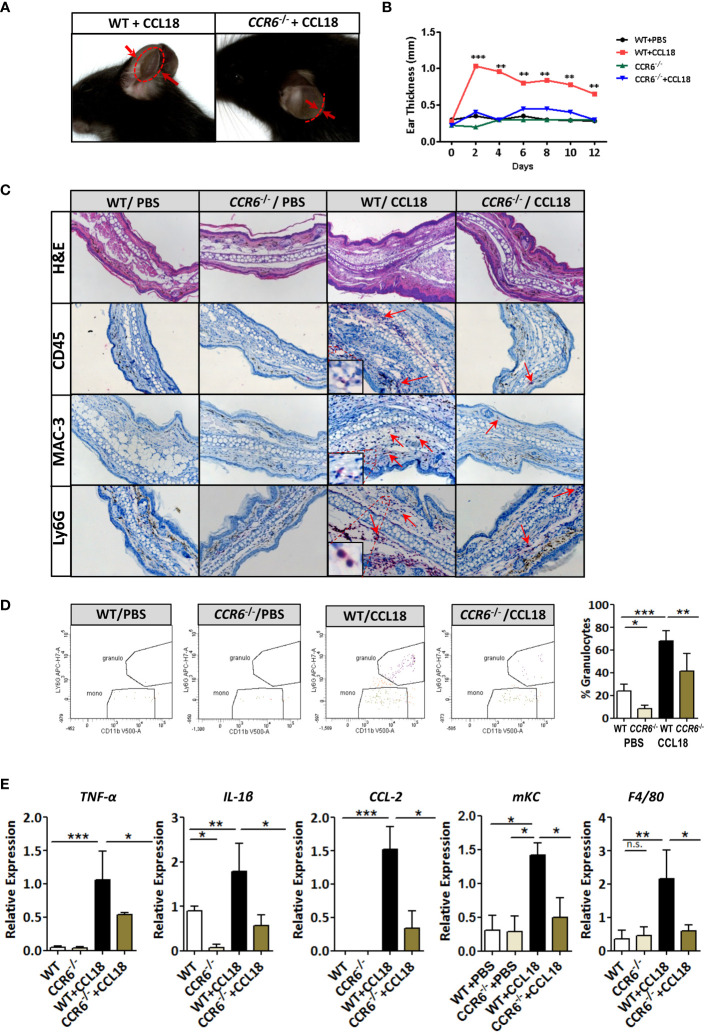
*CCR6* ^−/−^ mice are resistant to CCL18-induced leukocyte influx and inflammation. **(A)** Representative figure showing increased ear swelling after CCL18 administration to WT but not *CCR6* ^−/−^ mice. Arrows indicate ear thickness at point of maximal swelling (day 2). **(B)** Local CCL18 administration leads to sustained ear swelling. CCL18 (500 ng) or mock PBS was administered every other day for 12 days, by s.c. injection into the ears of WT (black and red symbols) versus *CCR6* ^−/−^ mice (green and blue symbols). Ear thickness was measured on non-injection days (n = 8–10 per group). **(C)** Early inflammation (day 2 after injection): representative HE-stained sections of PBS- and CCL18-injected ears from WT and *CCR6* ^−/−^ leukocyte infiltrates were visualized by IHC for CD45 (leukocytes), MAC3 (macrophages), and Ly6G (neutrophils). Arrows indicate positively stained cells. **(D)** Representative flow cytometry plots and quantitative data of granulocyte presence in CCL18- and PBS-injected ears of WT and *CCR6* ^−/−^. **(E)** TNFα, IL-1β, CCL2, and mKC *mRNA* expression in PBS- or CCL18-injected ears of WT and *CCR6* ^−/−^ (qPCR). F4/80 expression served as measure of macrophage accumulation. Values for **(D, E)** represent mean ± SEM of n = 8–10 mice per group; *P < 0.05, **P < 0.01, and ***P < 0.001; n.s., not significant.

Next, we studied the ability of CCL18 to drive CCR6-mediated responses *in vivo* in two models of inflammation: CCL18-dependent ear swelling and the more complex mouse model of atherosclerosis. In the ear inflammation model, CCL18-dependent inflammation was elicited by intradermal administration of this chemokine (vs PBS) (25). Ear thickness of CCL18-treated WT mice was progressively and significantly increased from day 2 onward, as compared with either *CCR6* ^−/−^ mice or PBS-treated WT controls (**Figures 5A, B**). Histopathological examination of ear tissue at early (day 2) and later stages of the inflammatory response (day 12) revealed clear features of acanthosis, increased keratin formation, vessel dilation, and dermal inflammatory infiltrates in CCL18- but not in PBS-treated ears in WT mice or CCL18-treated *CCR6* ^−/−^ mice (**Figure 5C**). The early time point was taken to allow assessment of late neutrophil and monocyte invasion as well as initial T-cell responses at the site of inflammation, considering that the CCL18 stimulus was provided every other day throughout the experiment. Infiltrates were analyzed by immunohistochemistry and flow cytometry (for gating strategy see **Supplementary Figure 3A**) to assess the composition of CCL18-induced inflammatory cell. CCD18-injected ears of WT mice were hallmarked by the abundant presence of CD45^+^ leukocytes. MAC-3^+^ and Ly6G^+^ cell infiltration in CCL18-treated ears of WT mice was significantly higher than that in *CCR6* ^−/−^ mice. Immunohistochemistry data were confirmed by flow cytometry, with markedly increased abundance of CD45+CD11c−CD11b−LyG+ cells, likely granulocytes in CCL18-treated ears of WT mice (**Figure 5D**). In addition, *mRNA* expression of pro-inflammatory cytokines such as TNF-α, IL-1β, CCL2, F4/80, and KC in ears of CCL18-treated *CCR6* ^−/−^ mice was significantly reduced compared to that of WT controls (**Figure 5E**). To further define the residual T cells infiltration in ear of *CCR6* ^−/−^ mice, we quantified CCR8 expression by qPCR (**Supplementary Figure 3B**), showing a non-significant increase of CCR8 expression in both WT and *CCR6* ^−/−^ inflamed ear after CCL18 stimulation (12).

### *CCR6* ^−/−^ mice do not accumulate CD4 T cells in response to intradermal CCL18 injection

**Figure 6:**
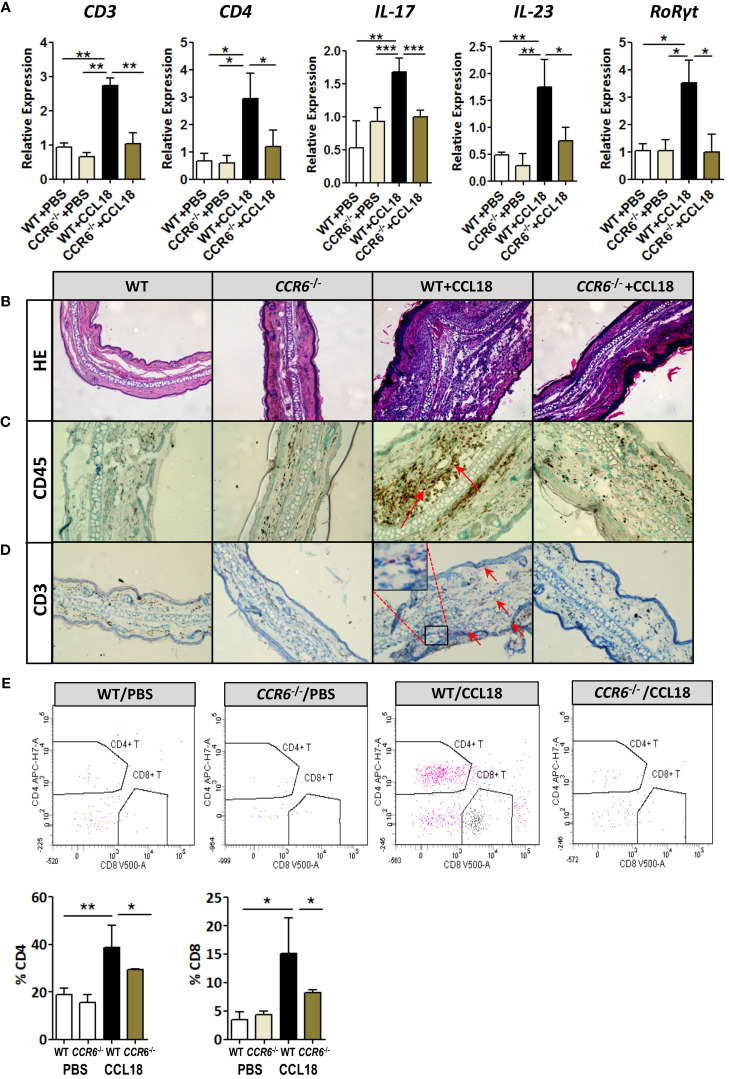
Th17-mediated skin inflammation following CCL18 treatment is ablated in *CCR6* ^−/−^ mice. **(A)** *mRNA* expression of CD3, CD4, and Th17 signature genes IL-17, IL-23, and RoRγt, respectively, in the mock- versus CCL18-challenged ears of WT and *CCR6* ^−/−^ mice (mean ± S.D. of n = 4–6 mice per group). **(B)** Chronic inflammation (day 12 after injection): Representative micrographs of H&E-stained sections of PBS- and CCL18-injected ears from WT and *CCR6* ^−/−^ mice. **(C)** Representative micrographs showing immunohistochemical staining for CD45 and CD3 demonstrating CD45^+^ leukocyte infiltration **(D)** and CD3^+^ T cells (both indicated by arrows). **(E)** Quantitative flow cytometry analysis of CD4^+^ and CD8^+^ T-cell infiltration into PBS- or CCL18-treated ears of WT and *CCR6* ^−/−^ mice. Quantitative values of relative T-cell subset abundance (% CD45^+^ parent population) are presented in the lower histograms. All values in panels **(B-E)** represent mean ± S.E.M. of n = 8–10 mice per group; *P < 0.05, **P < 0.01, and ***P < 0.001.

**Figure 7:**
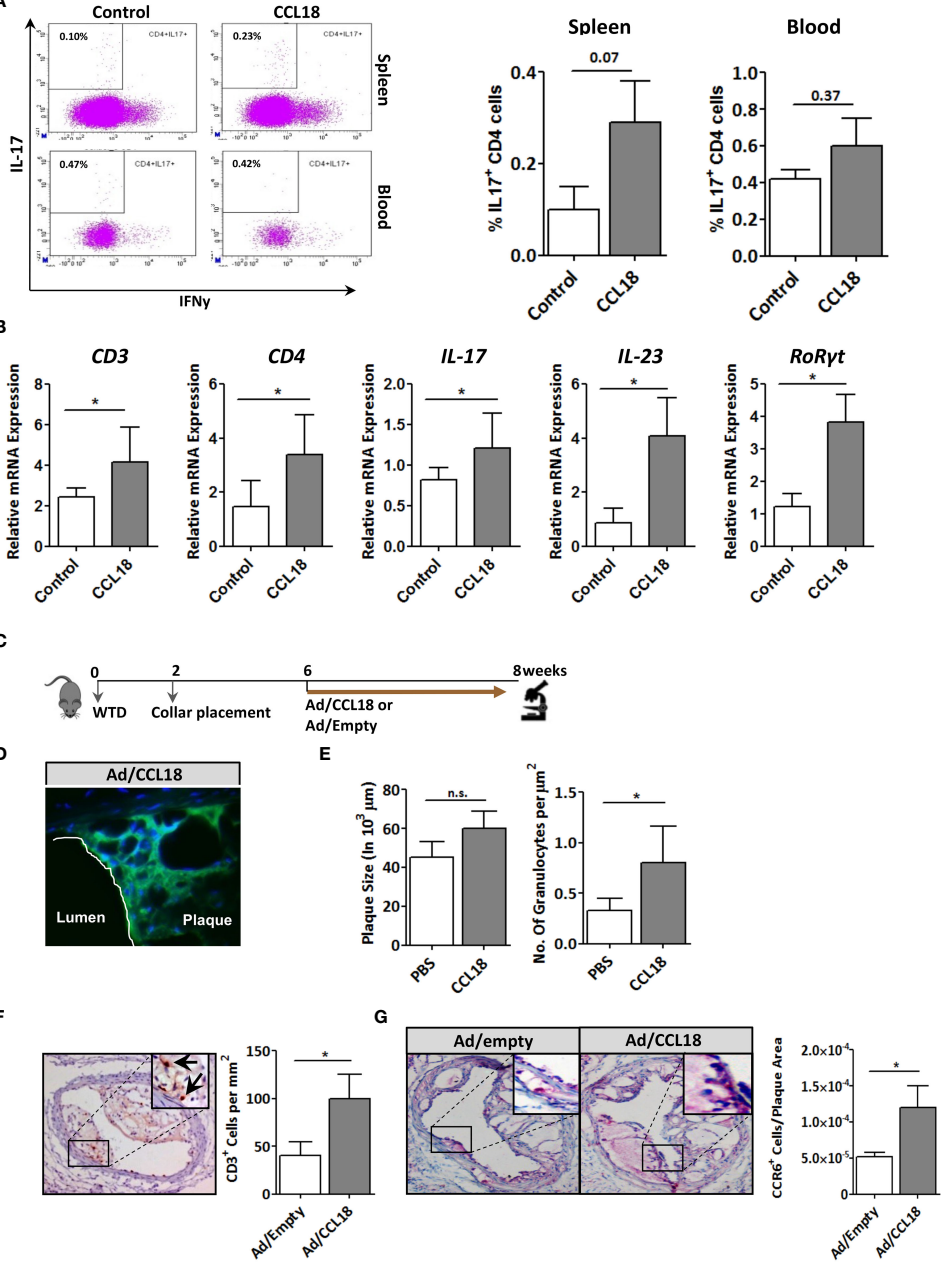
Involvement of the CCR6 axis in CCL18 pro-atherogenic activity. **(A)** Representative flow cytometry plots showing a trend toward increased presence of IL-17^+^ CD4^+^ T cells in spleen (P = 0.07; upper panels) but not blood (P = 0.37; lower panels) of PBS (control)– versus CCL18-treated *ApoE* ^−/−^ mice (CCL18). **(B)** Aorta expression of CD3, CD4, and Th17 signature genes (i.e., IL-17, IL-23, and RoRγt) in PBS- and CCL18-treated *ApoE* ^−/−^ mice (mean ± S.D; n = 4–6). **(C)** Scheme of the experimental setup of focal CCL18 gene transfer study in pre-existing plaque. **(D)** Successful CCL18 overexpression in carotid artery plaque after adenoviral gene transfer, as evidenced by abundant GFP reporter expression (green); DAPI was used as nuclear counterstain (40×). **(E)** CCL18 overexpression had no effect on plaque size but resulted in a significant increase in the number of granulocytes/µm2 in *ApoE^−/−^* mice **(F)** Local overexpression of CCL18 in the carotid plaque led to increased plaque CD3^+^ T-cell (CD3^+^ T cells are indicated by an arrow), and **(G)** CCR6^+^ cell content. Apart from panel **(B)**, all values represent mean ± SEM (n = 12); * P < 0.05; n.s., not significant.

CCR6 is a signature receptor of Th17 T cells (26). To determine the impact of prolonged CCL18 exposure on Th17 cell generation and recruitment, we monitored ear inflammation in WT or *CCR6* ^−/−^ mice for T-cell subset content and activation status by flow cytometry, immunohistochemistry (IHC), and qPCR at day 12. While at day 2 after injection, CD4^+^ T cells were undetectable in both PBS- and CCL18-treated WT mice by qPCR, IHC, and flow cytometry (data not shown); at day 12, *mRNA* expression of CD3^+^, CD4^+^, and CD8^+^, as well as Th17-related marker genes (e.g. IL-17, IL-23, and RoRγt) were significantly upregulated in ears of CCL18-treated WT mice compared with that of PBS-treated WT and to CCL18-treated *CCR6* ^−/−^ mice (**Figure 6A**). Interestingly, we also observed a minor population of CD8^hi^ T cells in several mice, which most likely represents an artefactual contamination derived from borderline gated non-TB cells, although we cannot include that they are terminally differentiated activated cytolytic T cells (27).⁠Ear inflammation was impaired in CCL18-treated *CCR6* ^−/−^ mice, as witness in the decreased leukocyte presence in stratum corneum and spinosum and in the dermal layer (**Figure 6B**). Notably, significantly higher numbers of CD45^+^ and CD3^+^ cells were observed in CCL18-treated ears of WT over *CCR6* ^−/−^ mice (**Figures 6C, D**). This was further corroborated by flow cytometry, with increments in aural CD4^+^ and CD8^+^ T-cell infiltration in WT versus *CCR6* ^−/−^ mice upon CCL18 treatment (**Figure 6E**). Further support for a CCL18-associated Th17 response was derived from flow cytometry analysis of T-cell composition of the spleen, blood, and lymph nodes in mice treated or not with CCL18. The IL17^+^ CD4^+^ T cells (Th17) abundance in the spleen tended to be increased, whereas the levels in blood (**Figure 7A**) and lymph node were unchanged (**Supplementary Figure 3C**). In support, aorta *mRNA* expression of CD3, CD4, IL-17, IL-23, and RoRγt was significantly upregulated in CCL18-treated compared to that in vehicle-treated *ApoE* ^−/−^ mice (**Figure 7B**), and similar increases were seen for spleen CD3, CD4, and IL17 expression (**Supplementary Figure 3D**). Collectively, the above results suggest that prolonged CCL18 exposure led to mobilization and/or skewing of T cells toward Th17.

### Validation of CCL18/CCR6 axis on atherosclerosis

Finally, we verified the relevance of the CCL18/CCR6 axis for plaque T-cell migration by focal overexpression of CCL18 in pre-existing plaques (for setup, see **Figure 7C**). Adenoviral infection of collar-induced carotid plaques was successful as judged from the pronounced GFP expression in plaque endothelium and central atheroma (**Figure 7D**). Brief CCL18 overexpression did not alter plaque size (**Figure 7E**), vascular smooth muscle cells (VMSC), macrophage, or collagen content (**Supplementary Figure 4**) or the frequency of internal elastic lamina ruptures (data not shown). No differences were seen in necrotic core size or Terminal deoxynucleotidyl transferase dUTPnick end labeling (TUNEL)-positive cell content (data not shown). However, CCL18 overexpression led to significantly increased plaque granulocyte (**Figure 7E**) and CD3^+^ contents, with an equivalent increase in CCR6^+^ cells, suggesting that T cells are a major CCL18-responsive subset in plaque (**Figures 7F, G**). The relevance of the CCR6/CCL18 axis for plaque T-cell migration was underpinned by an enrichment in CCR6^+^ cells in ruptured versus stable human carotid artery plaques (**Supplementary Figure 5A**). Moreover, the bulk of CCR6 staining colocalized with CD3^+^ T cells (**Supplementary Figure 5B**), and a tight correlation was observed between plaque CCL18 and CCR6 expression at *mRNA* level (**Supplementary Figure 5C**), lending further support to a link between CCR6 and CCL18 in atherosclerosis. Collectively, these findings suggest that the capacity of CCL18 to promote inflammation and destabilization of atherosclerotic cells is at least partly CCR6 dependent.

## Discussion

CCL18 expression has previously been detected in human atherosclerotic plaque and has been associated with future adverse cardiovascular events (8–10) but direct experimental evidence for a causal involvement in disease onset and progression is still lacking. Abundant expression of CCL18 in human carotid atherosclerotic plaque and particularly plaque macrophages has already been noted in 2008 by Hagg et al. (28). We confirm and extend these findings now showing that CCL18 expression increases with disease progression, colocalizes with plaque macrophages, and correlates with the pan-macrophage markers CD36 and the M2 marker CD163 (25). Moreover, we provide several lines of evidence that support a role of CCL18 in atherogenesis in a murine model of disease and identify CCR6 rather than CCR3 (23) or CCR8 (12) as a novel CCL18 receptor mediating T-cell recruitment. First, CCR6 co-immunoprecipitated with CCL18 in human and WT mouse T cells but not in CCR6 deficient T cells. Second, CCL18 stimulated a dose-dependent mobilization of Ca^2+^ in human Jurkat and mouse T cells but not in *CCR6* ^−/−^ T cells and induced similar receptor desensitization and internalization patterns as the canonical ligand CCL20. Third, the aforementioned *in vitro* data were corroborated *in vivo* by the ear swelling studies, indicating that CCL18 mediated leukocyte recruitment and that swelling is strictly dependent on CCR6. Concordant with this finding, focal overexpression of CCL18 led a significant enrichment in plaque CCR6^+^ T cells. Fourth, CCL18 administration in hyperlipidemic *CCR6* ^−/−^ mice neither did aggravate atherosclerosis nor did induce T-cell accumulation in plaque, unlike WT mice that rendered hyperlipidemic by PCSK9 gene transfer. If anything, CCL18-treated *CCR6* ^−/−^ mice even tended to show an inverse pattern, with reduced plaque burden and plaque T-cell content, potentially reflecting the residual contribution of CCR8. Finally, interaction of CCL18 and CCR6 was underpinned by *in silico* MD studies, revealing partly divergent domains of CCR6 to bind CCL20 versus CCL18. This opens opportunities for selective targeting of the CCL18 or the CCL20/CCR6 axis. Collectively, our findings implicate the CCL18/CCR6 axis in the pathogenesis of atherosclerosis.

The physiological relevance of the CCL18/CCR6 axis for focal inflammation and T-cell recruitment was apparent from both the focal plaque expression and ear swelling experiments. CCL18-treated WT mice featured accelerated temporal progression, presented with overt inflammatory myeloid infiltrates in early and T-cell accumulation in more advanced stages of disease, which were both not observed in *CCR6* ^−/−^ mice. Although our data do not allow drawing firm conclusions on the leukocyte influx dynamics in response to CCL18-mediated leukocyte recruitment, the IHC data and flow cytometry data seem to suggest that the CD4 T-cell influx is secondary to that of granulocytes (and, to a lesser extent, of macrophages), the dominate leukocyte subset in ear at day 2. Interestingly, whereas granulocyte influx was also lower in *CCR6* ^−/−^ mice, this subset only has very low expression of this receptor (as revealed by a search in Immgen databrowser). This raises the possibility that CCL18 first activates skin resident immune cells and most probably skin-associated B1 cells (although type 3 innate lymphoid cells (ILC3) or gamma/delta T cells may also contribute), which, in turn, triggers granulocyte recruitment. Further study is needed to pinpoint the responsible subset. Whereas CCL18 is regarded as a T-cell chemotactic (29), its counterpart, CCR6, is expressed by several T cells subsets, particularly Treg and Th17. CCL18-induced profound increases in plasma IL-6 were accompanied by IFNγ^+^ Th17 cell expansion in blood and spleen. In fact, IL-6 is implicated in Th17 differentiation, favoring a Foxp3^+^ Treg to pathogenic Th17 shift, in the presence of TGFβ (30). As the ear swelling model experiment also pointed to a Th17 signature, our data thus suggest a shifted Treg-to-Th17 balance, which may have impacted disease progression.

In summary, CCL18 exerts proatherogenic functions, through its binding partner CCR6, favoring the recruitment of CCR6^+^ T cells to sites of inflammation. CCL18/CCR6 may therefore be a candidate for intervention in atherosclerosis-related inflammation, albeit that our limited understanding of chemokine pathways relevant to human atherosclerosis, in mutual interaction, warrants further study.

## Data availability statement

Publicly available datasets were analyzed in this study. This data can be found here: GSE163154.

## Ethics statement

The studies involving humans were approved by Maastricht Medical Ethics Review Committee. The studies were conducted in accordance with the local legislation and institutional requirements. The human samples used in this study were acquired from primarily isolated as part of your previous study for which ethical approval was obtained. Written informed consent for participation was not required from the participants or the participants’ legal guardians/next of kin in accordance with the national legislation and institutional requirements. The animal studies were approved by Maastricht University Animal Ethics Committee. The studies were conducted in accordance with the local legislation and institutional requirements. Written informed consent was obtained from the owners for the participation of their animals in this study.

## Author contributions

AS: Formal analysis, Investigation, Methodology, Validation, Visualization, Writing – original draft. AK: Formal analysis, Investigation, Writing – review & editing. AC: Investigation, Writing – review & editing. KW: Investigation, Methodology, Writing – review & editing. LH: Investigation, Writing – review & editing. SJ: Investigation, Writing – review & editing. IB: Investigation, Writing – review & editing. SA: Resources, Writing – review & editing. TB: Supervision, Writing – review & editing. JWJ: Conceptualization, Supervision, Writing – review & editing. IC: Resources, Writing – review & editing. SM: Resources, Writing – review & editing. BM: Resources, Writing – review & editing. NN: Investigation, Writing – review & editing. GN: Investigation, Writing – review & editing. TH: Resources, Writing – review & editing. EL: Investigation, Writing – review & editing. FT: Conceptualization, Formal analysis, Writing – review & editing. EB: Conceptualization, Formal analysis, Funding acquisition, Resources, Supervision, Writing – review & editing.
