# Supplementary material for: CCL18 aggravates atherosclerosis by inducing CCR6-dependent T-cell influx and polarization
Source: Front Immunol. 2024 May 13;15:1327051. doi: 10.3389/fimmu.2024.1327051 (PMC11131369; doi:10.3389/fimmu.2024.1327051)

Supplementary Figure 1

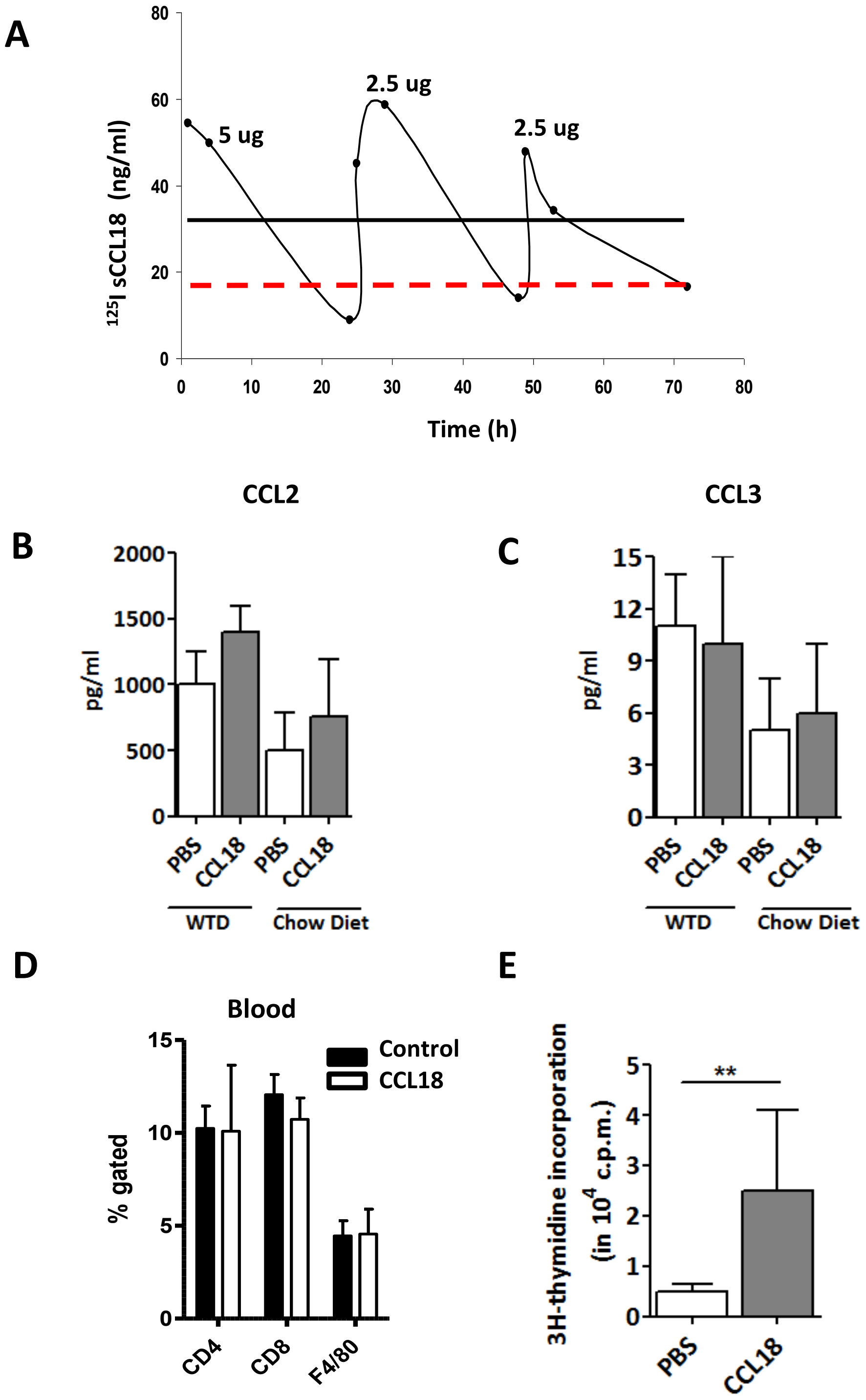

Supplementary Figure 2

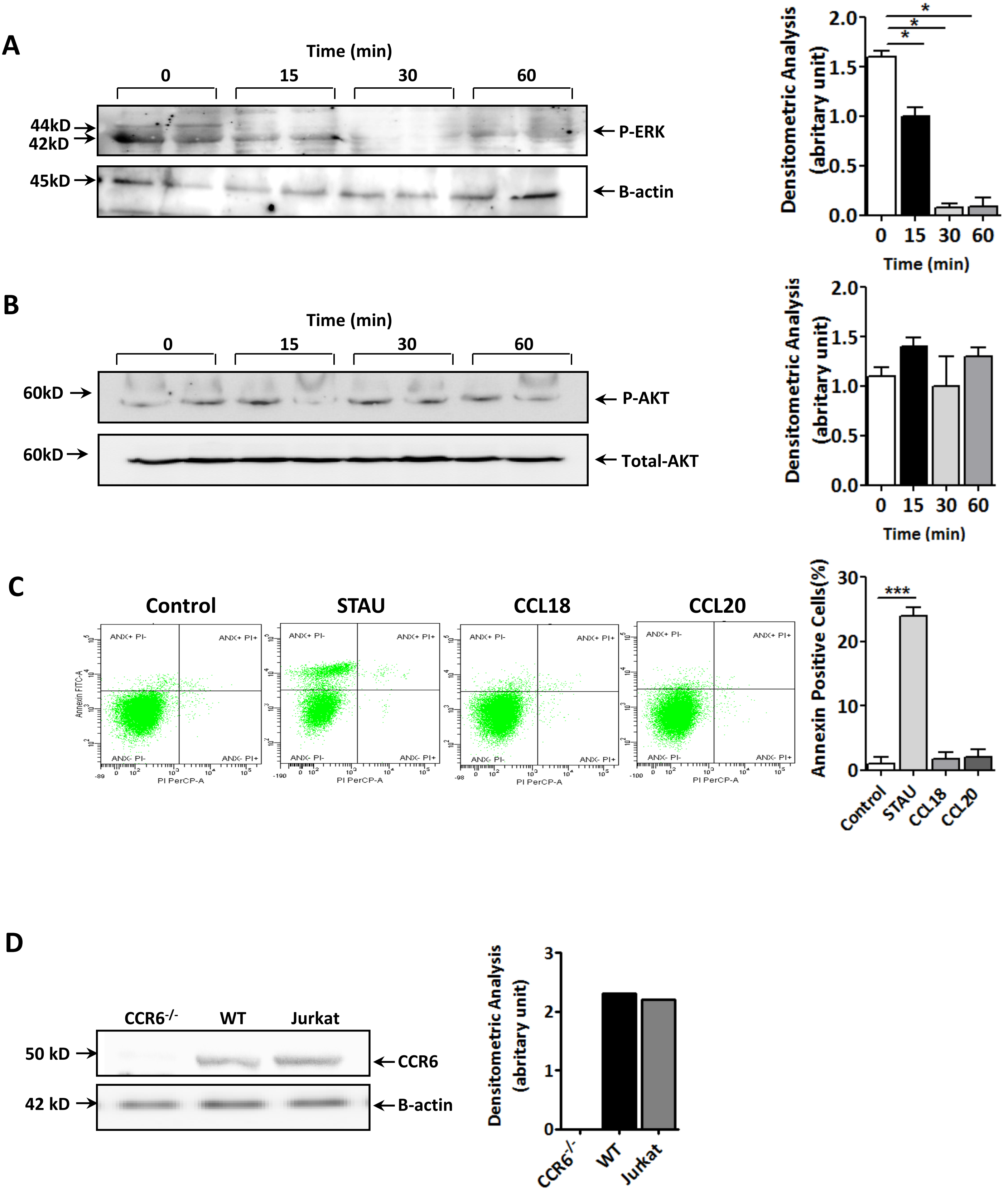

Supplementary Figure 3

A

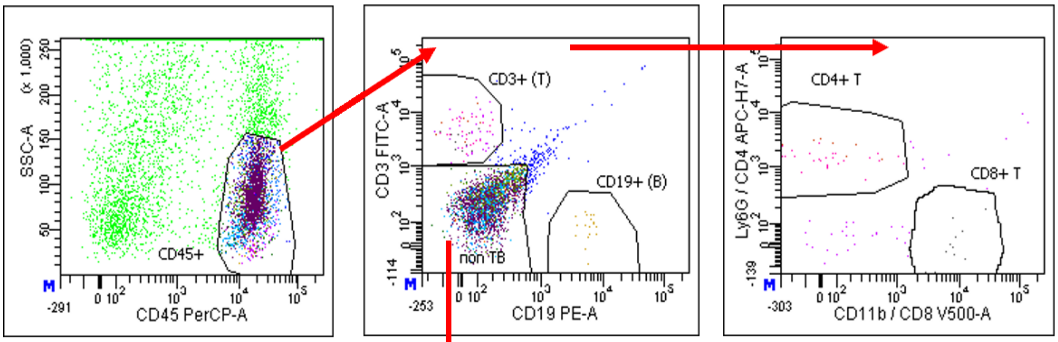
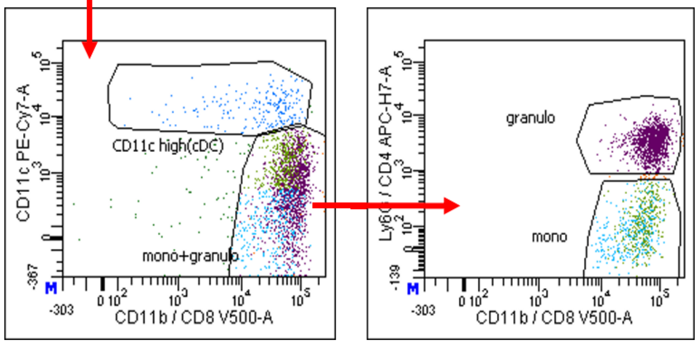

B

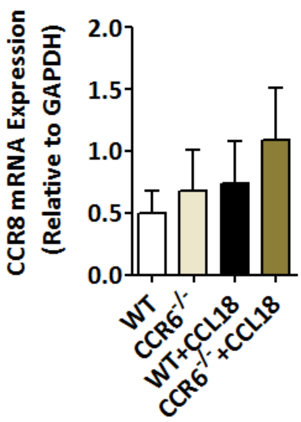

C

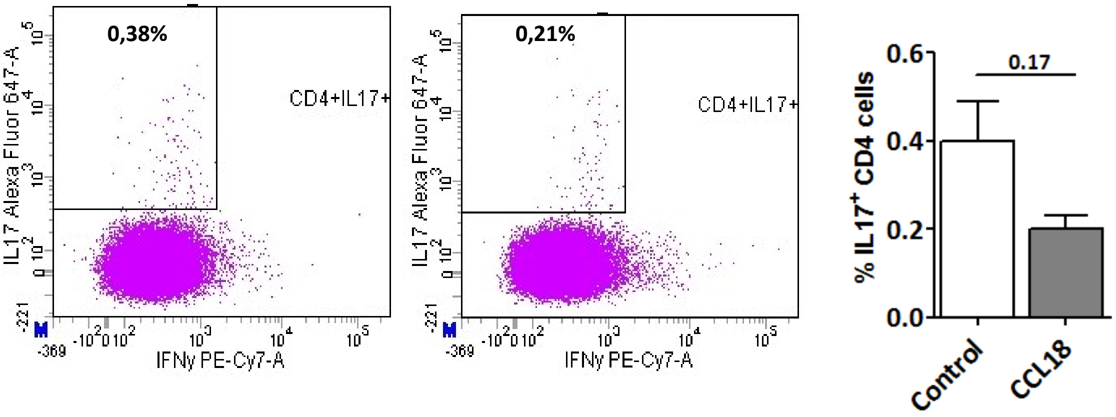

D

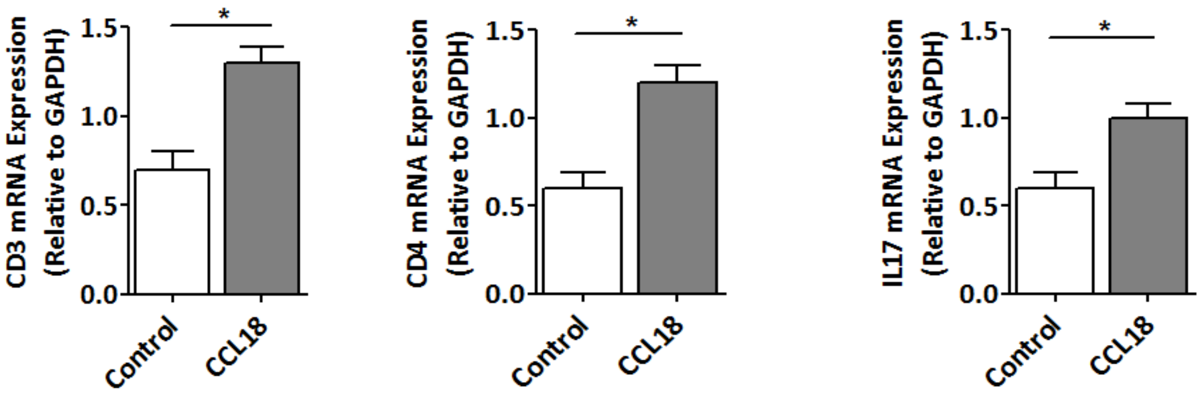

Supplementary Figure 4

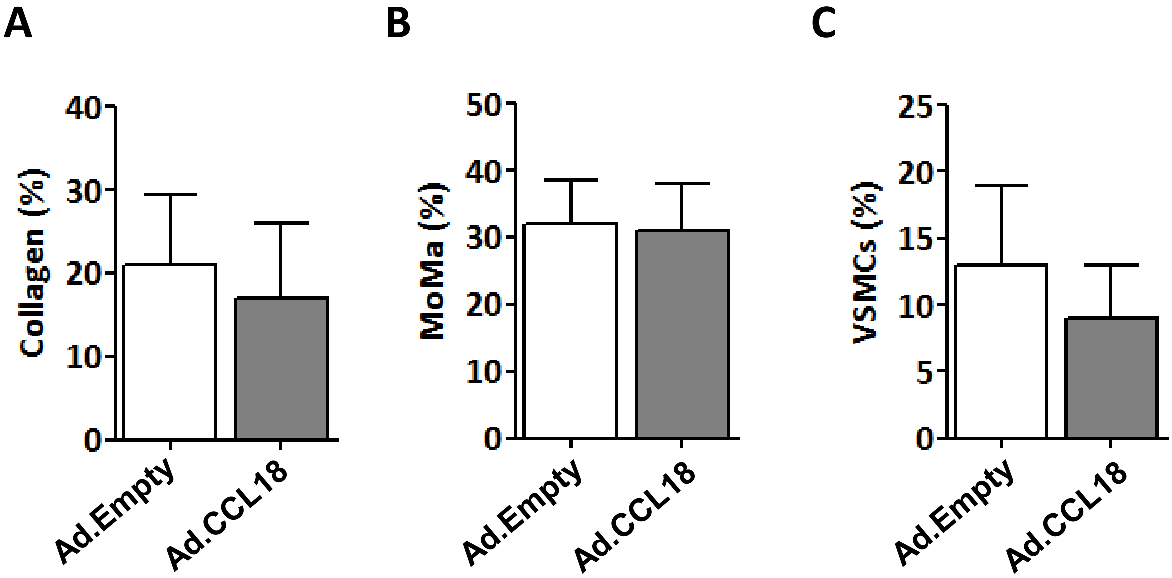

Supplementary Figure 5

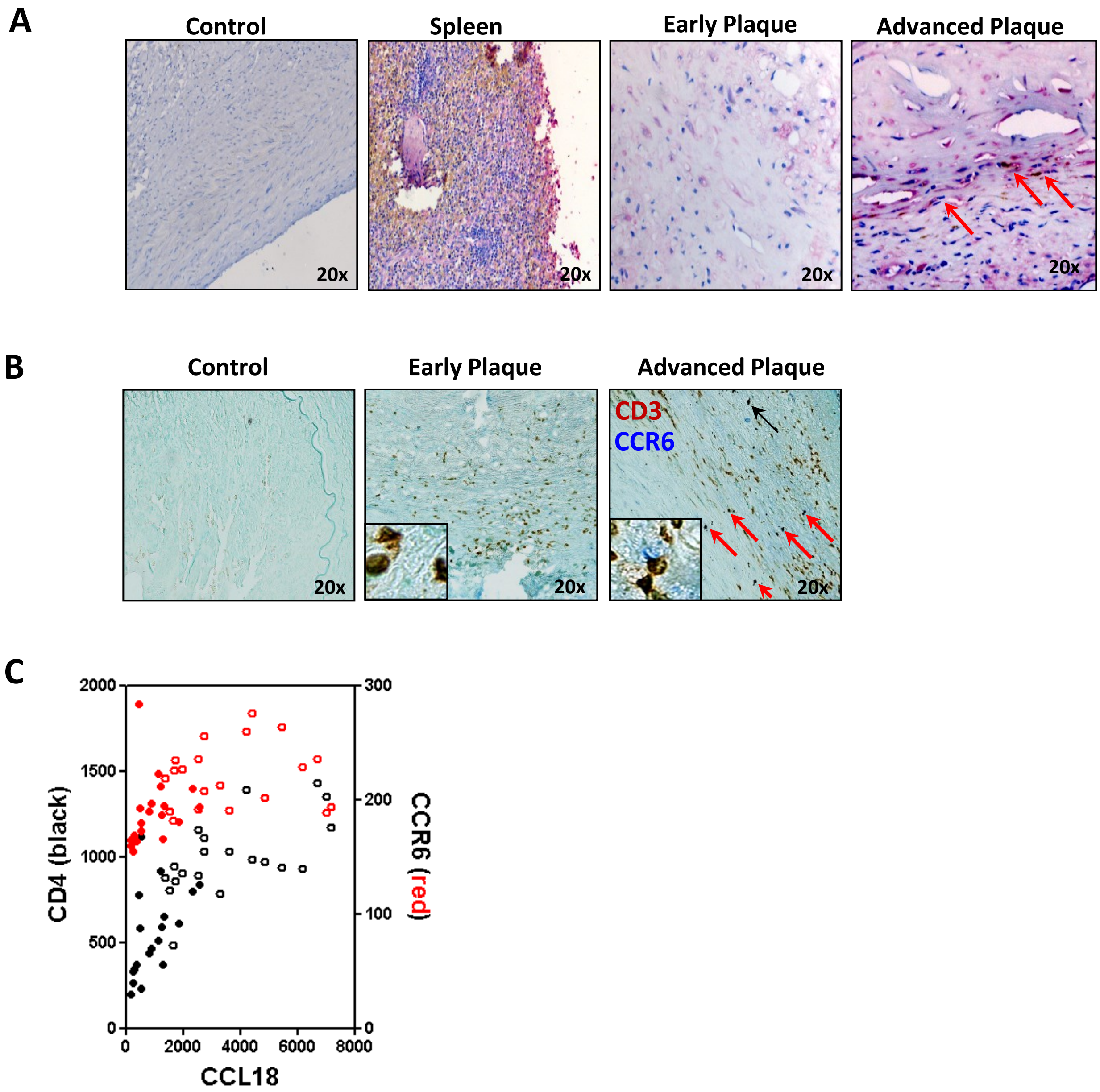

Supplement: Supplementary file 1 [file DataSheet_1.pdf]
