# Supplementary material for: CCL18 aggravates atherosclerosis by inducing CCR6-dependent T-cell influx and polarization
Source: Front Immunol. 2024 May 13;15:1327051. doi: 10.3389/fimmu.2024.1327051 (PMC11131369; doi:10.3389/fimmu.2024.1327051)
Supplement: Supplementary file 2 [file DataSheet_2.doc]

**Supplemental Methods**

**Human plaque expression studies**:

Total RNA was extracted from freshly frozen atherosclerotic tissue samples obtained from endarterectomy surgery (comparison advanced stable and advanced unstable (n=21) lesions; Department of Surgery, Maasland Hospital Sittard, Sittard, the Netherlands) or from autopsy (comparison early (n=13) and advanced stable (n=16) lesions; Department of Pathology, University Hospital Maastricht, Maastricht, the Netherlands). Collection, storage in the Maastricht Pathology Tissue Collection (MPTC) and patient data confidentiality as well as tissue usage were in accordance with the “Code for Proper Secondary Use of Human Tissue in the Netherlands” ([http://www.fmwv.nl](http://www.fmwv.nl/), <http://www.federa.org/sites/default/files/digital_version_first_part_code_of_conduct_in_uk_2011_12092012.pdf>). Tissue samples destined for RNA isolation were snap-frozen immediately after resection, staged by histological analysis of adjacent tissue sections according to Virmani *et al*.[1](http://www.nature.com/articles/srep15414#ref57) and grouped as advanced lesions (Tk/Tn FCA: thick or thin fibrous cap atheroma) or advanced unstable lesions (intraplaque hemorrhage or intramural thrombus). Lesions flanked at both sides by sections of a stable phenotype (based on morphological assessment) were considered to be stable and vice versa.

RNA was isolated with the Guanidine Thiocyanate (GTC)/CsCl gradient method and the NucleoSpin RNA II kit (Macherey-Nagel GmbH & Co. KG). RNA concentration was determined using the Nanodrop ND-1000 (Thermo Scientific) and quality was assessed by RNA 6000 Nano/Pico LabChip (Agilent 2100 Bioanalyzer, Palo Alto, CA, USA) analysis based on RIN (RNA integration number) values. RIN values above 5.6 were considered acceptable. Samples from autopsy were individually hybridized to HGU133 2.0 Plus arrays (Affymetrix, Santa Clara, USA, California); surgery samples were analyzed by Illumina Human Sentrix-8 V2.0 BeadChip® (Illumina Inc., San Diego, USA, California).

**RNA isolation of mouse tissue and cells**

RNA was isolated from mouse ear tissue by Trizol (Invitrogen, Carlsbad, CA) extraction. Approximately 50 mg of tissue was homogenized with 1 ml Trizol in a Tenbroeck homogenizer until no traces of tissue were visible. Following addition of 0.2 volume of chloroform, the suspension was centrifuged to separate the phases, the aqueous phase containing RNA was removed, and the RNA was precipitated with 0.5 volume of isopropanol and glycogen. Following two washes in cold 75% ethanol, the RNA pellet was allowed to air dry and re-suspended in RNAse free water.

**Animal studies**

All animal work, described below, was approved by the local regulatory authority of Maastricht University and in accordance with European and Dutch government laws and guidelines.

***In vivo* kinetics of CCL18**

CCL18 was radiolabeled with ^125^I (GE Healthcare, Diegem, Belgium) according to the iodogen method^2^. To examine its *in vivo* kinetics after repeated administration, ^125^I-CCL18 was administered once daily to C57Bl6 mice (Charles River, Maastricht, the Netherlands) by intra-peritoneal administration (doses of 2,5, and 5 μg); at 1, 4 and 24h after each injection blood samples were taken and counted for radioactivity. Mice were sacrificed at 72 h following the first injection, main organs and tissues were excised and ^125^I accumulation was counted by a Wizard 1470 automatic gamma counter (Perkin Elmer, Waltham, MA).

**Intradermal CCL18 injection**

We performed intradermal injection of 20 μl PBS, either alone or containing 500 ng recombinant mouse CCL18 (Serotech), into the ears of anesthetized mice using a 30-gauge needle every other day for 12 days. Ear thickness was measured before injection on day 0 and thereafter on non-injection days. Ear measurements were made using a caliper (GEM TOOL - Digital Thickness Gauge 0-25mm). All measurements were performed blinded. Mice were sacrificed at day 5 and day 12, and tissue was collected for histological, flow cytometric and expression analysis.

**Effect of systemic CCL18 administration on atherosclerosis**

Effects of systemic administration of CCL18 on pre-existing atherosclerosis was studied in ApoE^-/-^ mice (female; 10-12 weeks of age), fed with a Western-type diet containing 0.25% cholesterol and 15% cocoa butter (Special Diets Services, Witham, Essex, UK). Female mice were used as they show reduced variability in lesion formation compared to males. Diet and water were provided ad libitum. After 6 weeks of western-type diet, mice received an initiation bolus of CCL18 (5μg) in PBS/0.5%BSA (100μl) via intra-peritoneal injection, followed by two-daily injections of CCL18 (2.5μg) for two weeks. As controls, mice were injected with PBS/0.5%BSA (100μl). After isolation, the aortic arch and heart were fixed overnight in 1% PFA and paraffin-embedded sections (5 μm) were cut, starting from the commissure of the aortic cups upwards. Plaque size and necrotic core content in the aortic root were determined by morphometric analysis of the sections and averaging the mean lesion area of 10 consecutive sections by Leica QWin software.

**Focal overexpression of CCL18 in pre-existing plaques**

ApoE^-/-^ mice (n=24) (female, 10-12 weeks), were obtained from our own breeding stock and were put on a Western-type diet (WTD). After two weeks, mice received constrictive collars around both carotid arteries as previously described[^3^](#_ENREF_20). At this point collars were removed, the internal carotid artery as well as the common carotid proximal to the plaque were clamped and the adenovirus suspension (Ad.-CCL18-GFP and Ad-Empty-GFP, resp., at a titer of 1.0x10^10^pfu/ml; 20μl) was instilled via the left common carotid artery and left to incubate for 10’. Virus load for all treatment groups were equal and were tolerated well, with no detectable endothelial inflammatory effects. Two weeks after local incubation, the mice were sacrificed; before harvesting, the arterial bed was flushed for 10’ with PBS/p-formaldehyde (4%). Fixated carotid arteries were embedded in OCT (Sakura Finetek, Zoeterwoude, the Netherlands), snap frozen in liquid nitrogen and stored at -20°C until transverse cryosections were prepared (5µm).

**Recombinant AAV-PCSK-9 gene delivery into the WT and CCR6^-/-^ mice**

WT and CCR6^-/-^ littermates (females, 12-14 weeks of age) were injected intravenously with AAV-PCSK-9 (5x10^10^ p.f.u.) and challenged with WTD for six weeks[^4^](#_ENREF_5). After 6 weeks of WTD, WT and CCR6^-/-^ mice (n=6) received an initiation bolus of 5μg CCL18 in 100μl of PBS/0.5%BSA via intra-peritoneal injection, followed by two-daily injections of 2.5μg CCL18 for two weeks. As a control, mice were injected with 100μl of PBS/0.5%BSA. Blood samples were collected before and every other week after vector injection to monitor plasma cholesterol.

**Immunohistochemistry of human and mouse tissue**

Aortic root sections were stained for morphometric analysis with hematoxylin (Sigma Diagnostics) and eosin (Merck Diagnostica). Staining procedures for macrophages (MOMA-2), T cells (anti-CD3), TUNEL and collagen (Picro Sirius Red stain) were performed as previously described^5^.

For sections from paraffin-embedded whole ears (5 μm) were prepared by American Histolabs or HistoServe Inc. and stained with H&E. Paraffin ear tissue sections were deparaffinized by xylene and rehydrated. After pre-treatment with antigen retrieval sections were washed with TBS incubated with primary antibody overnight CD45 (Pharmingen clone; 30-F11), MAC-3 (Becton & Dickenson/ Pharmingen; clone M3/84), Ly6G (Becton & Dickenson). After overnight incubations sections were washed with TBS and incubated with a secondary biotinylated rabbit-anti-rat antibody (Dako Cytomation, Glostrup, Denmark). Thereafter, the sections were incubated with streptavidin ABC-alkaline phosphatase (Vector Laboratories) and color was developed with Vector red staining kit (Vector Laboratories) followed by haematoxylin counterstaining.

For human carotid artery immunohistochemistry, we first deparaffinized and rehydrated paraffin sections. After pre-treatment with pepsin buffer (1% in 0.1% HCL) for antigen retrieval, sections were incubated overnight at 4^0^C with primary rabbit anti-human CCL18 (Genwaybio) diluted in Tris buffered saline (TBS) containing 1% bovine serum albumin (BSA) and 0.1% Tween 20. After antigen retrieval sections were washed in TBS and incubated with a secondary antibody Swiss-anti-rabbit (Dako**)** biotinylated secondary antibody for CCL18. Thereafter, the sections were incubated with streptavidin ABC-alkaline phosphatase (Vector Laboratories, Burlingame, CA) and color was developed with Vector red (for macrophage costaining) and Vector blue (for VSMC, Vector Laboratories). For CCLL18 colocalization, macrophages were subsequently stained by anti-human CD68 (Dako-KP1, Dakocytomation) and vSMC by anti-human smooth muscle actin (Dako, clone: M0851), where CD68^+^ macrophages were counterstained with vector blue and ASMA^+^ VSMCs by DAB.

**Flow cytometry analysis of ear:** For flow cytometry analysis, mice were killed, ears collected, cut in small pieces, and digested in liberase TL (Roche Diagnostics GmbH, Mannheim, Germany) containing DNase (Roche Diagnostics GmbH, Mannheim, Germany) at 37°C for 45 minutes. Ear suspensions were passed through a 70 µm cell strainer (BD Biosciences, Franklin Lakes, NJ), centrifuged, resuspended and then immunostained with the following antibodies: CD45 PerCp (Biolegend / ITK; clone 30-F11), CD3 PE (ebioscience/ Bioconnect; clone 145-2C11), CD19 FITC (Becton & Dickenson), CD4 APCH7 (Becton & Dickenson; clone GK1.5), CD8 V500 (BD Pharmingen; clone 53-6.7), CCR6 BV421 (Biolegend/ ITK; clone 29-2L17), CD11b BV 510 (Biolegend; clone M1/70), Ly6G APCH7 (BD Pharmingen; clone 1A8), CD11c PECy7 (ebioscience/ Immunosource; N418).

**Intracellular staining for flow cytometry**

For Intracellular staining following antibodies used: CD3-BV510 (Biolegend/ ITK; clone 17A2), NK-1-1-pac Blue(Biolegend; clone PK136), CD11c- pac Blue(ebioscience/ Bioconnect), CD11b- pac Blue (ebioscience/ Immunosource; clone M1/70), CD19-efluo450 (ebioscience/ Immunosource; clone eBio1D3), CD4-APC-H7 (Becton & Dickenson; clone GK1.5), CCR6 (R & D; clone 140706), IL-17 Alexa 647 (Becton & Dickenson; clone TC11-18H10), IFNγ PerCy7 (BD Biosciences; clone XMG1.2), IL-10 FITC (BD Pharmingen; clone JES5-16E3).

### Immunoblot analysis

For Western blot analysis, protein extracts from Jurkat cells were subjected to 10% sodium dodecyl sulfate/polyacrylamide gel electrophoresis and blotted. Membranes (Whatman® Protran®) were probed with antibodies for P-AKT (Cell Signaling) P-ERK (Santa Cruz). As secondary antibodies, anti-rabbit-HRP (Cell Signaling) and anti-mouse-HRP (Santa Cruz) were used. Total AKT and ERK probing was performed as internal control using an antibody from (Cell Signaling; Santa Cruz).

**Cellular response on CCL18**

Jurkat T-cells were used to examine proliferation as well as gene expression after incubation with soluble CCL18. In brief, cells were plated in 24-well plates and grown to 70% confluence in Dulbecco’s Modified Eagle Medium (DMEM) supplemented with 10% fetal calf serum (FCS), 100 μg/ml streptomycin, 100U/ml penicillin and 2 mM L-glutamine. Afterwards, cells were serum starved in DMEM + 1% FCS for 10 hours and subsequently incubated for 16 hours with 30 ng/ml of CCL18, together with ^3^H-thymidine (925 GBq/mmol; Amersham, Uppsala, Sweden). The next day, ^3^H-thymidine incorporation was measured by a Packard 1500 liquid scintillation analyzer (PerkinElmer), and cells were harvested for RNA isolation. Guanidium thiocyanate-phenol was used to extract total RNA from cells and samples were subjected to DNAse I treatment (Promega, Madison, WS) after which cDNA was generated using RevertAid M-MuLV reverse transcriptase (Fermentas, Burlington, Canada) according to manufacturer’s protocol. Semi-quantitative gene expression analysis of IL-2, IL-6, IL-10, IL-15, CCR3, CCR5, CD-40, CD-69, TGF-β, IFN-γ, i-NOS, LXR and CYP-7α was performed using the SYBR-Green method on a FAST 7500 RT-PCR apparatus (Applied Biosystems, Foster City, CA).

**Migration assay**

Jurkat T-cells were stimulated with increasing concentrations (1-100 nM=7.9- 790 ng/ml) of CCL18 in a 96-well Neuroprobe plate (pore size 5 μm, Gaithersburg, MD). Chemotactic fMLP (1 nM, Sigma) was used as positive control, chemotaxis buffer (RPMI 1640, 25 mM HEPES and 0.1% BSA) as negative control. After 4 hours of incubation, pictures of the wells were taken and the migrated cells were counted manually.

**ELISA measurements**

Mouse serum derived from the systemic CCL18 experiment was used to determine circulating levels of IL-6, CCL2 (eBioscience) and CCL3 (Biosource, Nivelles, Belgium) according to manufactures protocol.

**Calcium Assay**

Cells were loaded with 2 µM Fluo-4 acetoxymethyl ester in the presence of Pluronic gel, basically as described[^6^](#_ENREF_4). After a wash step, the Fluo-4-loaded cells were resuspended at a concentration of 5 x 10^5^/ml, and agonist-induced changes in fluorescence intensity were determined in time using a thermostated SLM-Aminco spectrofluorometer under stirring. Changes in cytosolic [Ca^2+^]_i_ were expressed as pseudo-ratios F/F_o_, as described^6^.

### Molecular Dynamic (MD) simulations

The complexes between CCL3, CCL18 and CCL20 with CCR6 were determined by the application of protein-protein docking module implemented in ICM-Pro (version 3.4.6)^7^. These complexes were subsequently subjected to MD simulations using AMBER 12 program ^8^. Amber 99SB force field was applied for protein (CCLs and CCR), whereas Lipid11 force field[^9^](#_ENREF_23) was used for membrane (POPC). The complex was solvated by water (TIP3P model) within a radius of 12 Å from the molecular surface and counter ions were added to neutralize the system. To relax the system, we employed energy minimization after which position-restrained MD was carried out for 100 ps. Finally, free MD simulations were performed from 100 ps to 50 ns, while keeping the temperature and pressure constant at 300 K and 1 bar, respectively.

**Supplementary Figure Legends**

**Supplemental Figure 1:** **(A)** In vivo kinetic behaviour of ^125^I labelled CCL18 after repeated intraperitoneal injections. Serum levels gradually increase to approximately 17 ng/ml after three days (red dotted line); average serum levels were approx. 32 ng/ml (black line) **(B+C)** CCL18 treatment did neither alter CCL2 and CCL3 plasma levels in western fat diet (WTD) fed ApoE^-/-^ nor in chow fed WT mice compared to PBS treated controls. Mean ± S.E.M. of n=8. **(D)** Flow cytometric analysis of blood. In general, CCL18 did not influence macrophage and T-cell distribution patterns in both tissues; mean± S.E.M. of n=12. **(E)** CCL18 induced proliferation determined by [^3^H]-thymidine incorporation with Jurkat cells; mean ± S.D. of n=4; ** P<0.01.

**Supplemental Figure 2:** Western blotting for phosphorylated and total ERK **(A)** and AKT **(B)** of CCL20 (30ng/ml) stimulated human Jurkat T cells as a function of time after stimulus. Quantification of the Western blot bands by densitometry. Mean ± S.D. of n=3-4 **(C)** Apoptosis in human Jurkat T cells determined by Annexin-A5 flow cytometry. Jurkat T cells stimulated with CCL18 (100 ng/ml) and CCL20 (30 ng/ml). Staurosporine (STAU; 300nM) used as a positive control. Values are presented as mean ± S.E.M. of n=4; ** P<*0.05, ****P<*0.001. **(D)** Western blotting for CCR6 confirmed efficient CCR6 deletion in CCR6^-/-^ mice. Β-Actin served as loading control. Quantification of the Western blot bands was done by densitometry.

**Supplemental Figure 3: (A)** Scheme of the gating strategy for the ear FACS study (Day 2 and 12) **(B)** mRNA expression of CCR8 in ear of WT or CCR6^-/-^ mice 2 days after intradermal CCL18 administration. **(C)** IL17^+^ CD4^+^ T cell abundance within the total CD3+ T cell population in lymph nodes tended to be decreased in CCL18 treated ApoE^-/-^ mice compared to PBS treated controls **(D)** Spleen CD3, CD4 and IL17 mRNA levels were determined by qPCR in spleen of ApoE^-/-^ and CCL18 treated ApoE^-/-^ mice. Values are presented as mean ± S.E.M. n= 5-6); ** P<*0.05.

**Supplemental Figure 4:** Local adenoviral overexpression of CCL18 in carotid artery plaque did neither result in differences in plaque collagen content **(A),** nor in MOMA-2^+^ macrophage **(B) or** ASMA^+^ VSMC content **(C)**.

**Supplemental Figure 5:** **(A)** Immunohistochemistry for CCR6 in stable advanced and advanced unstable human carotid artery atherosclerotic lesion (n=6), power 20x **(B)** Co-localization of CCR6 (blue) and CD4 (brown), n=6 (magnification power 20x) **(C)** Correlation analysis of CCL18 expression in human atherosclerotic plaque indicated a significant association with CCR6 and CD4 expression. Values are presented as mean ± S.E.M., ** P<*0.05; n.s.: not significant.

**References**

1. Virmani, R., Kolodgie, F. D., Burke, A. P., Farb, A. & Schwartz, S. M. Lessons from sudden coronary death: a comprehensive morphological classification scheme for atherosclerotic lesions*.* *Arteriosclerosis, thrombosis, and vascular biology* 20, 1262–1275 (2000).

2. Sobal G, Resch U and Sinzinger H. Modification of low-density lipoprotein by different radioiodination methods. *Nuclear medicine and biology*. 2004;31:381-8.

3. von der Thusen JH, van Berkel TJ and Biessen EA. Induction of rapid atherogenesis by perivascular carotid collar placement in apolipoprotein E-deficient and low-density lipoprotein receptor-deficient mice. *Circulation*. 2001;103:1164-70.

4. Bjorklund MM, Hollensen AK, Hagensen MK, Dagnaes-Hansen F, Christoffersen C, Mikkelsen JG and Bentzon JF. Induction of atherosclerosis in mice and hamsters without germline genetic engineering. *Circulation research*. 2014;114:1684-9.

5. de Nooijer R, Verkleij CJ, von der Thusen JH, Jukema JW, van der Wall EE, van Berkel TJ, Baker AH and Biessen EA. Lesional overexpression of matrix metalloproteinase-9 promotes intraplaque hemorrhage in advanced lesions but not at earlier stages of atherogenesis. *Arterioscler Thromb Vasc Biol*. 2006;26:340-6.

6. van Kruchten R, Mattheij NJ, Saunders C, Feijge MA, Swieringa F, Wolfs JL, Collins PW, Heemskerk JW and Bevers EM. Both TMEM^16^F-dependent and TMEM16F-independent pathways contribute to phosphatidylserine exposure in platelet apoptosis and platelet activation. *Blood*. 2013;121:1850-7.

7. Abagyan R and Totrov M. Biased probability Monte Carlo conformational searches and electrostatic calculations for peptides and proteins. *Journal of molecular biology*. 1994;235:983-1002.

8. Steinbrecher T, Latzer J and Case DA. Revised AMBER parameters for bioorganic phosphates. *Journal of chemical theory and computation*. 2012;8:4405-4412.

9. Skjevik AA, Madej BD, Walker RC and Teigen K. LIPID11: a modular framework for lipid simulations using amber. *The journal of physical chemistry B*. 2012;116:11124-36.
